# Stimulation of hERG1 channel activity promotes a calcium-dependent degradation of cyclin E2, but not cyclin E1, in breast cancer cells

**DOI:** 10.18632/oncotarget.2829

**Published:** 2015-01-19

**Authors:** Mathew Perez-Neut, Andrew Shum, Bruce D. Cuevas, Richard Miller, Saverio Gentile

**Affiliations:** ^1^ Department of Molecular Pharmacology and Therapeutics, Loyola University Chicago, Maywood, IL, USA; ^2^ Department of Pharmacology, Northwestern University, Chicago, IL, USA

**Keywords:** Cyclin E2, hERG1, breast cancer, calcium-dependent degradation

## Abstract

Cyclin E2 gene amplification, but not cyclin E1, has been recently defined as marker for poor prognosis in breast cancer, and appears to play a major role in proliferation and therapeutic resistance in several breast cancer cells. Our laboratory has previously reported that stimulation of the hERG1 potassium channel with selective activators led to down-regulation of cyclin E2 in breast cancer cells. In this work, we demonstrate that stimulation of hERG1 promotes an ubiquitin-proteasome-dependent degradation of cyclin E2 in multiple breast cancer cell lines representing Luminal A, HER2+ and Trastuzumab-resistant breast cancer cells. In addition we have also reveal that hERG1 stimulation induces an increase in intracellular calcium that is required for cyclin E2 degradation. This novel function for hERG1 activity was specific for cyclin E2, as cyclins A, B, D E1 were unaltered by the treatment.

Our results reveal a novel mechanism by which hERG1 activation impacts the tumor marker cyclin E2 that is independent of cyclin E1, and suggest a potential therapeutic use for hERG1 channel activators.

## INTRODUCTION

Type-E cyclins are encoded by two distinct genes (CCNE1 and CCNE2) that play a major role in promoting transition from G0/G1 to S phase of the cell cycle [1–3]. Although both E cyclins have been associated with mechanisms of tumor progression, only the CCNE2 gene has been reported to be preferentially amplified in different types of breast tumors [4–6]. Although reported to be undetectable in non-transformed cells [7], overexpression of cyclin E2 in breast cancer cells has been found to be associated with increased resistance to both chemotherapy and endocrine therapy [8, 9], and thus cyclin E2 has been included in the gene expression pattern that predict poor prognosis in endocrine-resistant and metastatic breast cancers. Despite the relevance of cyclin E2 to breast cancer biology, the vast majority of studies defining the regulatory mechanisms that control type-E cyclins has been dedicated only to cyclin E1.

Cyclin E1 shares high homology (46% identity) and important functional domains with cyclin E2, including a binding site for the E3 ligase Fbw7 [10]. During the S phase of the cell cycle, GSK-3β-dependent phosphorylation of cyclin E1 plays a major role in promoting recognition and ubiquitination of cyclin E1 by the ubiquitin ligase Fbw7. Consequently, ubiquitinated cyclin E1 is degraded via the ubiquitin-proteasome pathway [10, 11]. However, the mechanisms that control cyclin E2 turnover are less well established.

Potassium ion channels have been traditionally known for their role in neurons and muscles as they regulate membrane potential and developments of action potential [12]. However, recent investigations have shown that, potassium channels are involved in cell cycle control and that the hERG1 potassium channel expression level can vary during the cell cycle of non-excitable cancer. This suggests that hERG1 may play a fundamental role in the biology of cancer [13–15].

It has also been shown that, stimulation of potassium channels leads to hyperpolarization of the membrane [16, 17] which in turn results in an increased driving force for passive calcium entry. However, the role of hERG1 potassium channel in controlling calcium homeostasis and proliferation in cancer cells remains unexplored. Furthermore, it is very well known that changes in calcium homeostasis can control cell faith however, understand how calcium signaling functions in specific cellular processes including proliferation it is still challenge.

We have previously demonstrated that, chronic stimulation of hERG1 potassium channels leads to an accumulation of the breast cancer cells in the G0/G1 phase of the cell cycle [14]. We have also shown that, several proteins known to play an important function in the transition from G0/G1 phase were down-regulated by exposing cells to hERG1 channel activator NS1643 for 24 hours. In contrast, we observed that cyclin E2 protein level was significantly reduced after only 2 hours of drug treatment, indicating that acute hERG1 channel activation inhibits cyclin E2 protein expression in a manner that is distinct from the chronic effects exerted on other cell cycle regulators. However, the mechanism by NS1643 induces cyclin E2 inhibition has not been established. In the present work we have investigated on the mechanism through which stimulation of hERG1 channel leads to a rapid down-regulation of cyclin E2, assessed the impact of this inhibition in tumor cells arising from a variety of breast cancer subtypes, and considered the potential of hERG1 activators in treating breast cancers that have become resistant to therapy.

## RESULTS

### Stimulation of hERG1 channel activity selectively down-regulates cyclin E2 protein level

We have previously shown that, acute stimulation of hERG1 ion channels in breast cancer cells promotes reduction (60%) of cyclin E2 protein level, whereas chronic stimulation down-regulated other cell cycle regulators [14]. This suggests that hERG1 stimulation exerts a rapid control on cyclin E2 activity and, by extension, cell cycle progression.

**Figure 1:**
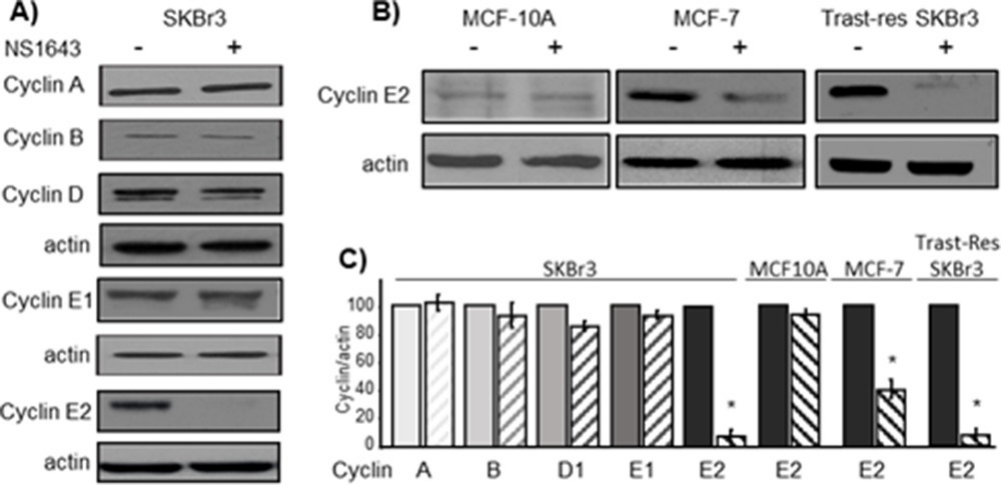
NS1643-induced hERG1 channel activity selectively inhibits cyclin E2 in breast cancer cells SKBr3 breast cancer cells were exposed to NS1643 (50 mM) for 4 hr. Cell lysates were resolved by SDS-PAGE. **(A)** Immunoblot analysis of cyclins in lysates from cells treated with or without NS1643. β-actin served as a loading control. Due to extraneous antibody stripping of membrane, the same lysates were re-probed for cyclin D, E1 or E2. **(B)** Representative cyclin E2 immunoblots showing effect of NS1643 treatment on SKBr3 cells, a SKBr3 cell line selected for Trastuzumab-resistance, and MCF7 cells. **(C)** Bar graphs summarizing the observed effects of NS1643 exposure on cyclin protein expression (*n* = 3; *p < 0.01).

To further investigate the short-term effects of stimulation of hREG1 channel we monitored the effects of NS1643 on different types of cyclins in various breast cancer cell lines, including ER-negative/Her2-positive (SKBr3) or ER-positive cells (MCF-7). Interestingly, we found that, stimulation of hERG1 channel led to a rapid reduction of cyclin E2 protein level (Figure 1), but none of the other cyclins protein level including cyclin E1, A, B and D changed upon application of NS1643 for 4 hr (Figure 1A, 1C, Supplementary Figure 1 and Supplementary Figure 2). In addition, application of NS1643 to a non-transformed breast cell line (MCF10A) did not have any effect either on cyclin E2 (Figure 1B) or cell proliferation rate (data not shown) suggesting that the effect of NS1643 is specific to hERG1 positive cancer cells (Supplementary Figure 1).

Recent investigations have revealed that, amplification of cyclin E2 is strongly associated with Trastuzumab resistance and poor prognosis. Interestingly, our experiments showed that stimulation of hERG1 channel dramatically reduced cyclin E2 protein level in Trastuzumab-resistant SKBr3 cells as well (Figure 1B).

**Figure 2:**
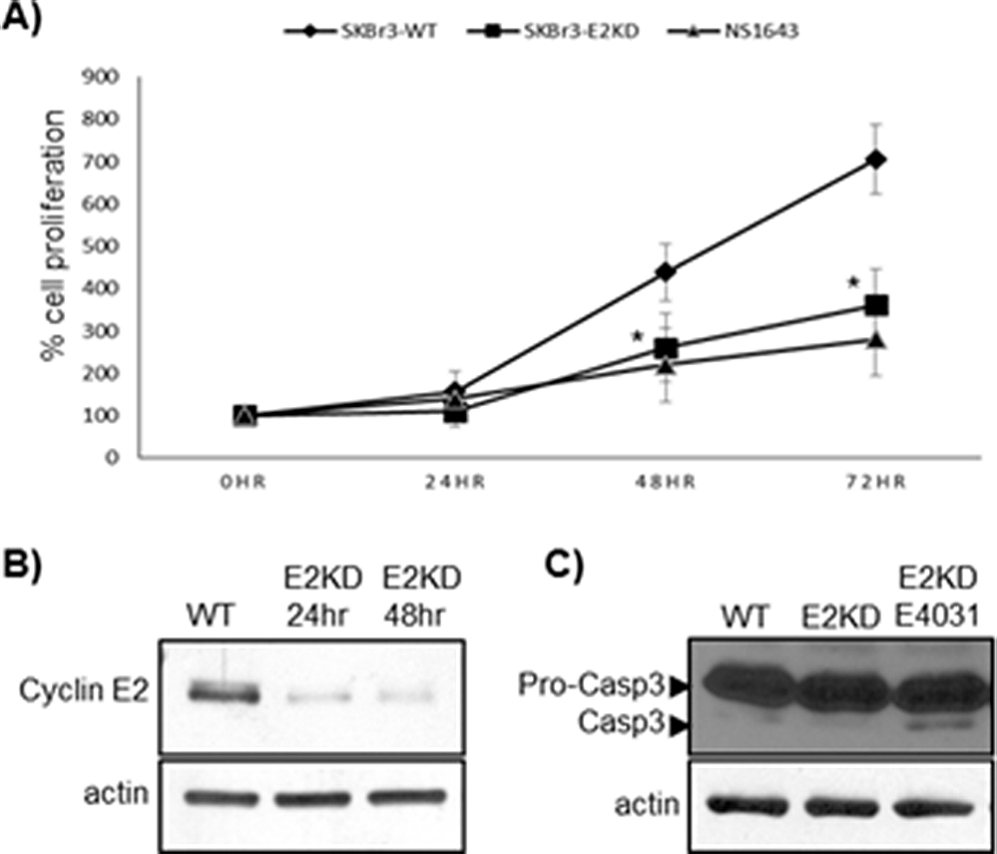
Suppression of cyclin E2 inhibits proliferation of SKBr3 breast cancer cells Cyclin E2 expression was knocked down by transfecting SKBr3 cells with cyclin E2-specific siRNA oligonucleotides. **(A)** Graph comparing the cell number (obtained by cell counting) over time of SKBr3 cells transfected siRNA targeting cyclin E2 (SKBr3-E2KD) compared to non-transfected SKBr3 cells (WT) cells or with SKBr3 cells treated with NS1643 (*n* = 6; *p < 0.01). **(B)** Immunoblot analysis showing efficacy of siRNA-mediated cyclin E2 knockdown in SKBr3 cells. **(C)** A representative anti-caspase 3 immunoblot showing the relative caspase 3 cleavage in cells transfected with cyclin E2 siRNA alone, treated with hERG1 channel antagonist E4031 (control; to induce apoptosis [28]) and non-transfected cells (top panel). In both (B) and (C), actin immunoblots are utilized as loading controls (lower panels).

Next, we assessed the effect of suppression of cyclin E2 on cell proliferation. Knocking down cyclin E2 by its siRNA strongly inhibited proliferation in SKBr3 breast cancer cells (Figure 2A, 2B) compared to wild type SKBr3 cells. Similar to the inhibitory effect of NS1643 on SKBr3 cell proliferation (Figure 2A), this event was not accompanied by an increased cleavage of procaspase-3 (Figure 2C) suggesting that the reduction of cell proliferation was independent from cell death.

Taken together with our previous report, these data indicate that the inhibitory effect of NS1643 on cyclin E2 expression is not limited to a single breast cell line.

### NS1643-dependent down-regulation of cyclin E2 is mediated by the ubiquitin-proteasome pathway

**Figure 3:**
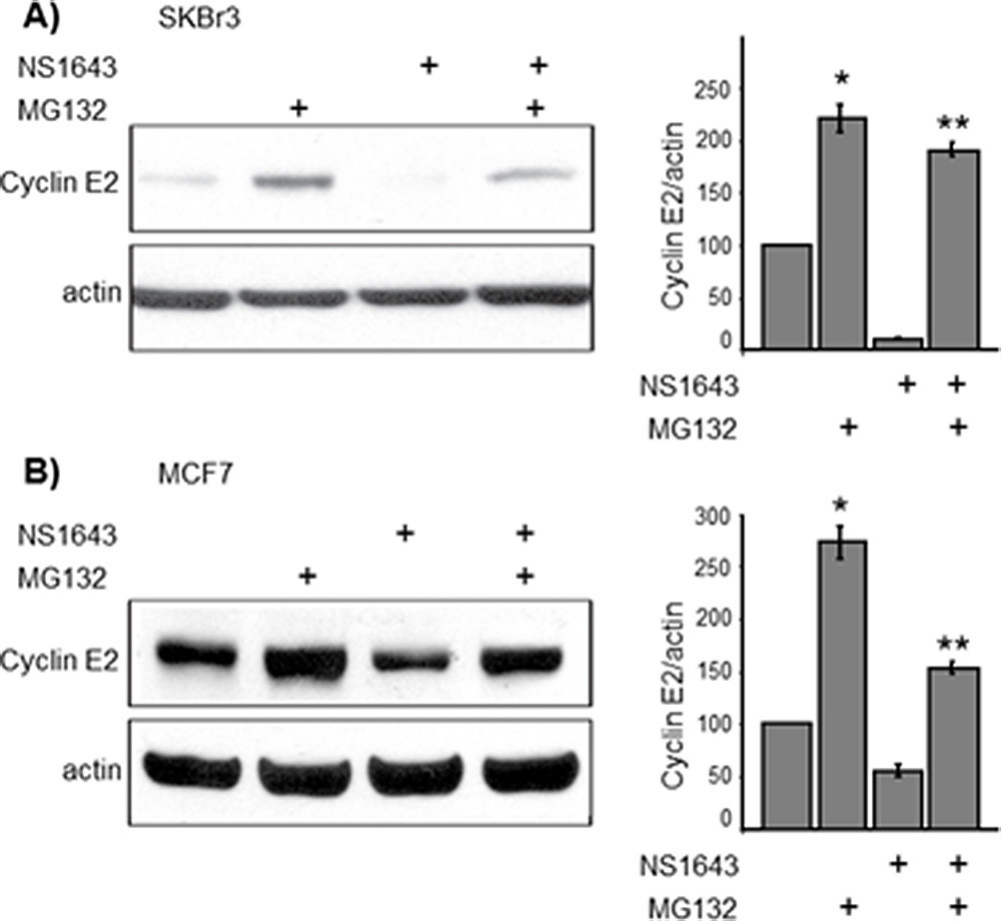
NS1643-induced hERG1 channel activity promotes proteasome-dependent degradation of cyclin E2 (**A** and **B** cyclin E2 immunoblot analysis in SKBr3 and MCF7 breast cancer cells exposed to the proteasome inhibitor MG132 (10 mM) alone, NS1643 (50 mM) alone or MG132 + NS1643 for 4 hr (upper panels). In both **(A)** and **(B)**, bar graphs summarize the impact of NS1643 and MG132 on cyclin E2 protein as assessed by densitometry analysis of immunoblots from multiple experiments (*n* = 4; *p < 0.01; ** *p* < 0.01).

**Figure 4:**
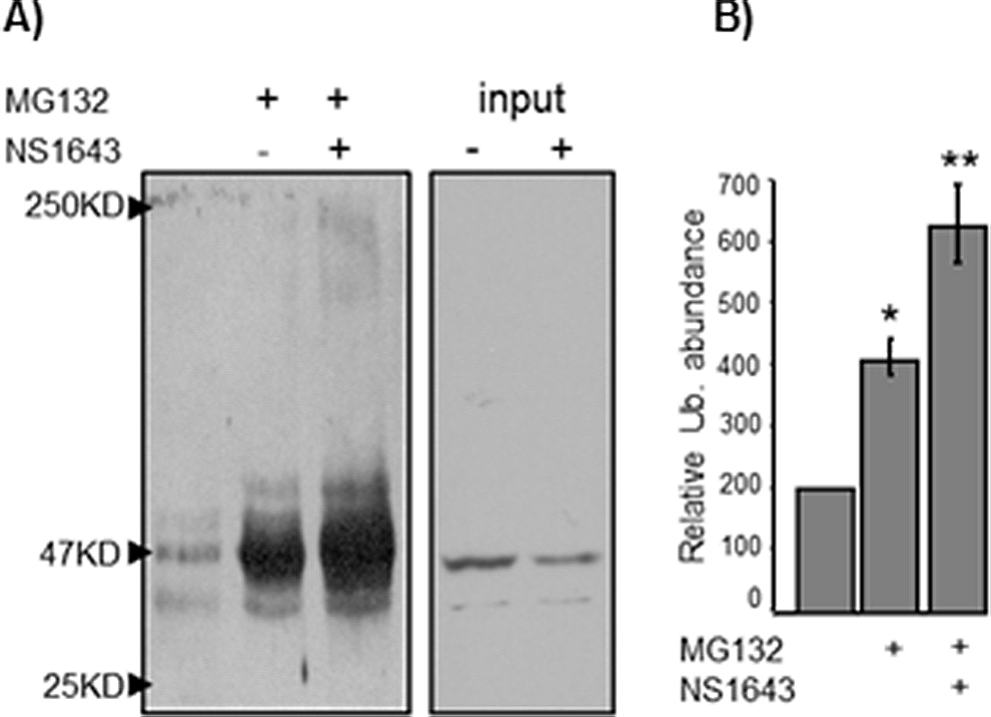
NS1643-induced hERG1 channel activity induces cyclin E2 ubiquitination SKBr3 breast cancer cells were exposed to MG132 alone or MG132 + NS1643. Endogenous cyclin E2 was immunoprecipitated using anti-cyclin E2 antibodies, and immunoprecipitates were resolved by SDS-PAGE and immunblotted with anti-ubiquitin antibodies. **(A)** A representative anti-ubiquitin immunoblot of immunoprecipitated cyclin E2. **(B)** Graph depicts the combined densitometry analysis of three independent experiments. (*p < 0.01, **p < 0.01).

It has been previously reported that type-E cyclins can be degraded via the ubiquitin-proteasome pathway [11]. Furthermore, cyclin E1 turnover can be mediated by recognition of phosphodegron motif by the ligase E3 Fbw7 [10]. This recognition motif is conserved in cyclin E2, but cyclin E2 has not been shown to be regulated by this mechanism. Therefore, we asked whether NS1643 treatment induced proteasomal degradation of cyclin E2. We blocked proteasome-dependent degradation with proteasome inhibitor MG132 in both SKBr3 and MCF7 cells and utilized anti-cyclin E2 immunoblot analysis to assess the impact of this blockade on cyclin E2 expression (Figure 3). We found that the proteasome inhibitor enhanced cyclin E2 protein expression compared to untreated cells confirming that cyclin E2 can be degraded via the proteasome. Interestingly, MG132 strongly inhibited the effect of NS1643 on cyclin E2, suggesting that that stimulation of hERG1 channel promotes degradation of cyclin E2 via the proteasome. To determine whether stimulation of hERG channels induces ubiquitination of cyclin E2, endogenous cyclin E2 was immunoprecipitated from cells treated with MG132 alone or MG132 + NS1643 and then immunoblotted with anti-ubiquitin antibodies. We discovered that the presence of NS1643 strongly increased ubiquitination of cyclin E2 (Figure 4). Taken together, our data strongly suggest that stimulation of hERG1 channel leads to degradation of cyclin E2 via activation of the ubiquitin-proteasome pathway.

It has been reported that Glycogen synthase kinase 3 beta (GSK3-β) is an important regulator of ubiquitin-dependent degradation of E-types cyclins [18]. Therefore, to assess whether GSK3-β played a role in NS1643-dependent degradation of cyclin E2, we monitored the activity of this kinase in cells treated with NS1643 for 4 hours. Phosphorylation of GSK3-β on serine residue 9 is associated with markedly reduced kinase activity, therefore we utilized immunoblot analysis with phospho-specific antibodies as a surrogate assay of GSK3-β activity. Interestingly, we found that stimulation of hERG1 channel with NS1643 was associated with increased GSK3-β phosphorylation on serine 9, consistent with reduced kinase activity within the time frame associated with cyclin E2 degradation (Supplementary Figure 3). Altogether, our data suggests that stimulation of the hERG1 channel in breast cancer cells activates ubiquitin-proteasome-dependent degradation of cyclin E2 that is independent of GSK3-β.

### NS1643-dependent degradation of cyclin E2 is calcium dependent

**Figure 5:**
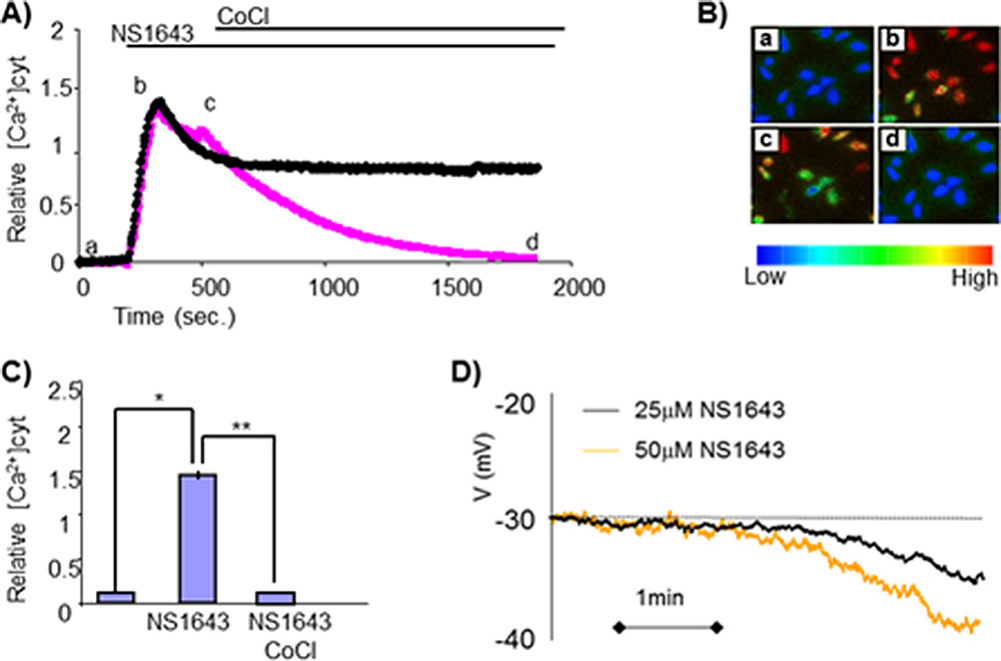
Sustained elevation in intracellular Calcium after treatment with NS1643 **(A)** Changes in cytosolic calcium levels as measured by Fura-2 calcium imaging in SKBr3 cells before and after application of NS1643 or NS1643 followed by 10 mM CoCl2. **(B)** Pseudocolored images showing relative levels of Calcium concentration (blue = low calcium; red = high calcium) were visualized by Fura-2 340 nm/380 nm fluorescence at different time points a) basal calcium density; b) peak of calcium density, c) calcium level at the time of CoCl2 application, d) calcium level after CoCl2 application. **(C)** Histograms summarizing the effect of NS1643 compared to control on intracellular calcium levels before (NS1643) and after application of CoCl2 (NS1643 + CoCl2) (*n* = 46; * < 0.001; ** < 0.001) **(D)** Continuous records made under zero current clamp conditions showing the hyperpolarizing effects of different concentrations of NS1643 on the SKBr3 membrane potential (Vm).

**Figure 6:**
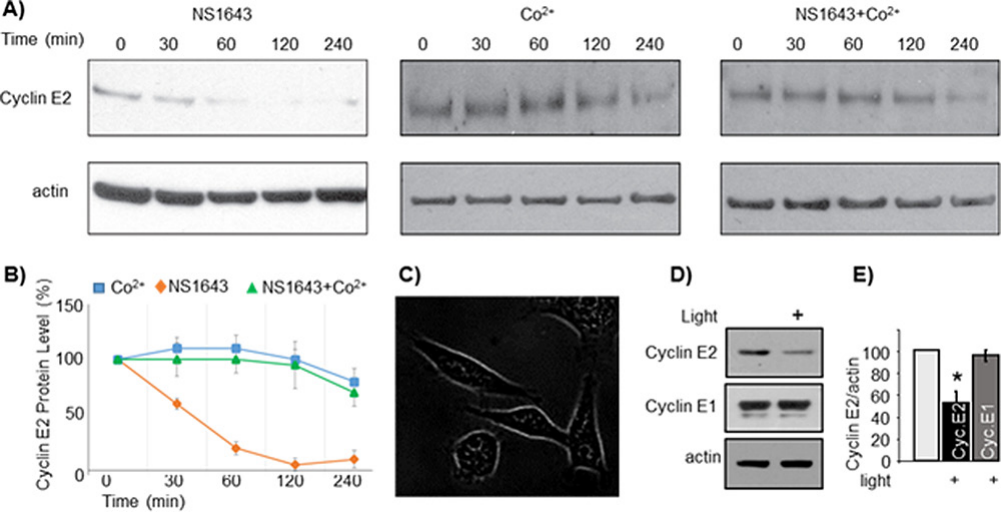
NS1643-dependent degradation of cyclin E2 is modulated by calcium entry **(A)** Representative immunoblots from SKBr3 cells exposed to NS1643 alone (50 uM), cobalt chloride alone (10 uM) or NS1643 + CoCl2. Cell lysates were resolved by SDS-PAGE and immunblotted with anti cyclin E2 antibodies (top panels) and anti actin antibodies (lower panels). **(B)** Graphical depiction of the relative protein abundance over time as determined by the immunoblot analysis in (A) **(C)** SKBr3 cells expressing a DNA construct encoding for channelrhodopsin-2 conjugated with GFP (ChR2-GFP). Image was obtained with fluorescence microscopy. **(D)** Representative immunoblots of SKBr3-ChR2 cells lysates. Cells were either exposed to blue light (470 nm) or maintained in darkness for four hours prior to lysis. **(E)** Graph depicts the combined densitometry analysis of three independent light-induced degradation experiments (*p < 0.01).

The E3 ubiquitin ligases mediate specific recognition of target proteins, yet [19] the large abundance of members (> 1000) in the E3 ligase protein family makes the identification of specific E3 that targets a substrate protein very challenging. Among the diverse family of E3 ligases, a subset have been shown to be sensitive to intracellular calcium concentrations, which can stimulate E3 ligase activity directly [20, 21] or indirectly [22]. Interestingly, in non-excitable cells (such as breast cancer cells), an increased calcium entry can be achieved by activation of potassium channels [17]. Therefore, we tested the hypothesis that, stimulation of hERG1 activity could promote calcium influx in breast cancer cells. Utilizing a whole-cell current clamp approach, we found that application of the hERG1 channel activator NS1643 promoted hyperpolarization of the SKBr3 membrane potential (Figure 5B), suggesting that his event could provide the driving force for calcium entry. We then utilized Fura-2 fluorescence to detect NS1643-induced changes in intracellular calcium, and discovered that 1643 treatment induced a dramatic increase of intracellular Ca2+ in breast cancer cells (Figure 5A, 5C, 5D). This event was completely reversed by application of the generic calcium channel blocker cobalt (CoCl2, Figure 5A). Having established that NS1643 treatment increases intracellular calcium levels, we investigated on the role of hERG1-driven calcium entry on cyclin E2 degradation. Strikingly, we found that the effect of NS1643 on cyclin E2 was completely abolished by blocking calcium entry (Figure 6A, 6B), suggesting that calcium concentration is a crucial regulator of NS1643-induced cyclin E2 degradation.

To separate calcium-dependent cyclin E2 degradation from that induced by calcium-independent consequences of NS1643 treatment, we designed a novel approach to initiate controlled influx of extracellular calcium without use of chemicals or ionophores. Channelrhodopsin-2 (ChR2) is a light-gated, membrane-bound cation channel derived from algae that selectively allows calcium entry upon activation by exposure to 470 nm wavelength light [23–25]. We first stably expressed ChR2 in SKBr3 breast cancer cells (Figure 6C) then we induced calcium entry by exposing the cells to 470 nm light under usual culture conditions (37°C, 5% CO2).

We found that stimulation of ChR2 led to a significant decrease of cyclin E2 (Figure 6D, 6E), but not cyclin E1, protein levels. This data suggests that, NS1643-induced ubiquitin-dependent down-regulation of cyclin E2 is calcium-dependent, and that degradation of cyclin E2 can be regulated by calcium influx.

**Figure 7:**
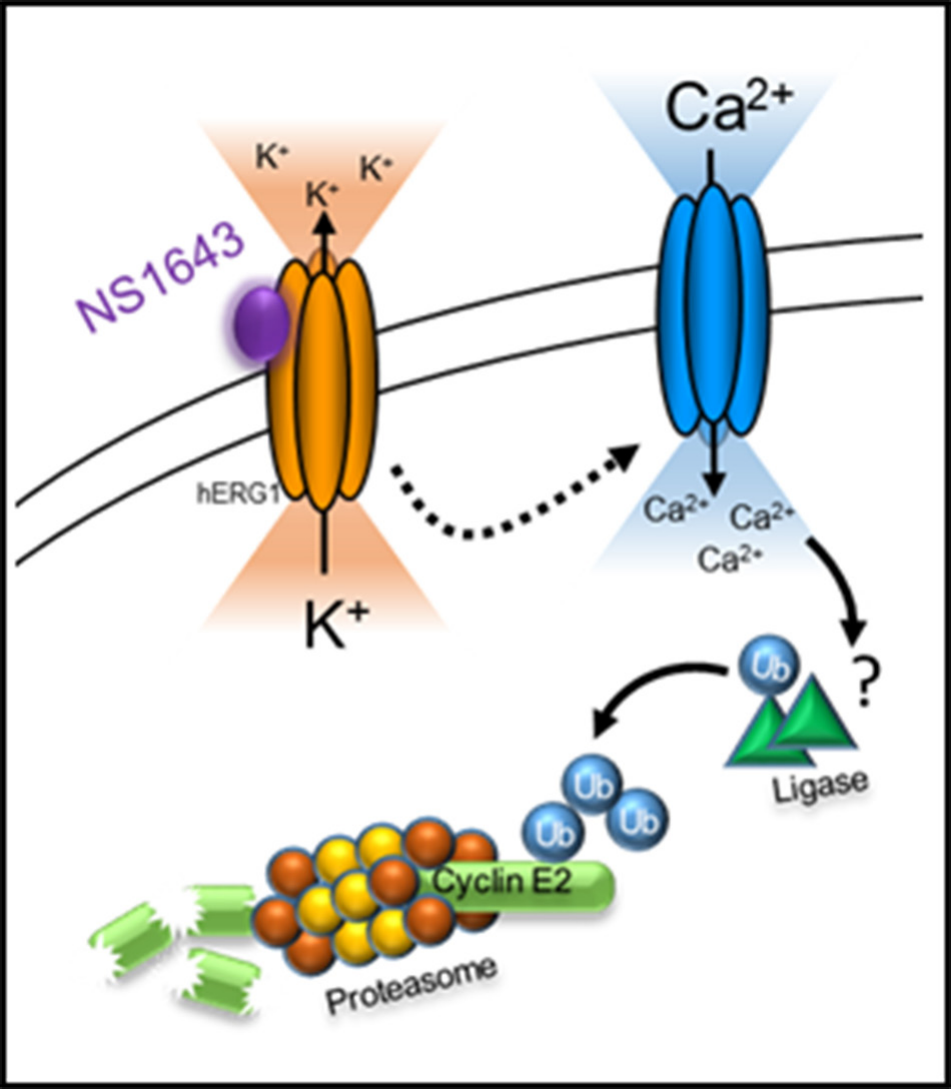
Schematic representation of hERG1 activator-dependent degradation of cyclin E2 Stimulation of hERG1 potassium channel with NS1643 leads to increased passive calcium entry due to hyperpolarization of the cell. Increased intracellular calcium promotes the ubiquitination and consequent proteasome-dependent degradation of cyclin E2.

Altogether, our findings strongly suggest that stimulation of the hERG1 potassium channels in breast cancer cells induces a calcium-dependent degradation of cyclin E2 via activation of an ubiquitin-dependent proteasome pathway (Figure 7).

## DISCUSSION

Targeting ion channels as a strategy for anticancer therapeutics has been proposed, but few studies have addressed the feasibility of this approach as of this date.

Potassium channels such as the hERG1 protein, that shows enhanced expression in several cancers of different origins and/or stages may represent therapeutic targets if, manipulation of activity of such channels could inhibit tumor cell proliferation. To test this hypothesis, in our previous studies we examined the impact of hERG1 activation in breast cancer cell biology. We were the first to show that stimulation of the hERG1 potassium channel in breast cancer cells promoted down-regulation of several proteins that play important role for the advancement through the different phases of the cell cycle, and that these changes were associated with the induction of cellular senescence. In addition to the effects of chronic NS1643 administration, we also observed that acute exposure to NS1643 induced rapid loss of cyclin E2 expression. However, we did not investigate the mechanisms controlling cyclin E2 expression in that work.

In this study, we have determined that the functional consequence of acute hERG1 stimulation on cyclin E2 occurs by activation of a calcium-dependent degradation of cyclin E2 by activating an ubiquitin-proteasome pathway.

Calcium ion is a universal signaling factor that controls several cellular processes including proliferation, secretion, motility or death. To keep calcium homeostasis under strict control, cells use a variety of systems that regulate activity of calcium transporters. Previous investigations have demonstrated that increased intracellular net negative charge (hyperpolarization) in non-excitable cells (e.g. melanomas; lymphocytes) determined an electrochemical driving force for calcium (positive charge) resulting in increased calcium entry. Interestingly, in our experiments the loss of intracellular potassium obtained by stimulation of hERG1 with a selective agonist (NS1643) leads to cell hyperpolarization and increase in intracellular calcium. These experiments for the first time provide evidence that changes in activity of the hERG1 channel in cancer cells can control calcium homeostasis. However, the current status of the understanding the role of hERG1 and/or other potassium channels in controlling calcium homeostasis during the cell proliferation (e.g. different phases of the cell cycle) needs further investigation.

Calcium can cross the surface membrane via activation of voltage-gated calcium channel (VGCC). However, hyperpolarization inhibits the activity of these channels therefore we exclude that a possibly expressed VGCC in breast or other cancer cell types could be responsible for the NS1643-dependent calcium entry.

However, increased intracellular calcium can often occur as a consequence of a biological process in which calcium passing across the surface membrane is able to activate calcium release from intracellular stores. This event occurs by stimulation of ryanodine receptors (calcium induced calcium release; CICR). In addition, intracellular stores can also express calcium channels that can be activated by other ligands such as Inositol-3-phospate (IP3). Our experiments were not designed to test the contribution of ligand-gated ion channels in regulating the effects of NS1643. However, application of the generic surface membrane calcium channel blocker CoCl2 completely and rapidly restored intracellular calcium level in the presence of NS1643. This suggests that NS1643 do not affects IP3-dependent signaling. To understand the role of ryanodine receptor more experiments need to be performed.

At this time, we cannot exclude the contribution of other ion channels such as those that are indirectly regulated by depletion of intracellular calcium stores (ORAIs). However, our experiments suggest that, NS1643-dependent increased calcium entry is most probably mediated by activity of passive calcium transporters (e.g. TRPV channels).

Ubiquitination plays a major role for regulating protein function in many cellular processes such as cell division. The attachment of ubiquitin to targeted proteins is the final step of the ubiquitination cascade that leads to protein degradation via the proteasome.

Interestingly, our experiments demonstrated that, NS1643-dependent changes in intracellular calcium led to degradation of cyclin E2 via activation of an ubiquitin-proteasome system. This suggests that stimulation of hERG1 channel activates an E3 ligase. The E3 ligase protein family presents wide heterogeneity among its several hundred members. Therefore, identifying a specific E3 ligase targeting cyclin E2 upon stimulation of hERG1 channel it is very challenging. However, our experiments suggest that calcium could regulate the E3 ligase downstream of hERG1 activated pathway. Interestingly, still few E3 ligases have been reported to be controlled by calcium such as Nedd4 family of E3 ubiquitine ligase. Identification of the possible E3 ligase(s) that can control calcium-dependent degradation of cyclin E2 will be the focus of our future studies.

Interestingly, among E-type cyclins only cyclin E2 is degraded upon stimulation of hERG1 channel. This is surprising, as cyclin E1 and cyclin E2 share high homology (46% identity) and many important functional domains that have been demonstrated to be involved in the regulation of type E functions and degradation.

For example, it has been shown that GSK3β-dependent phosphorylation of cyclin E1 controls the binding of the E3 ligase Fbw7 to cyclin E1, resulting in cyclin E1 degradation via the ubiquitin-proteasome pathway. Since the phosphodegron for Fbw7 identified in cyclin E1 is also present on cyclin E2, it has been hypothesized that degradation of cyclin E2 occurs by a similar mechanism as for cyclin E1. However, Fbw7-dependent degradation of cyclin E2 has not been established.

In addition, in contrast to the canonical GSK3β-dependent mechanism for cyclin E1 degradation, our experiments revealed that NS1643 treatment induces GSK3β phosphorylation that is consistent with reduced kinase activity suggesting that GSK3β might play different roles in the degradation processes of cyclin E2 that are not necessarily linked to an increase of its kinase activity.

In conclusion, although the ubiquitin-proteasome system appears to play a major role in the NS1643-induced degradation of cyclin E2, our data strongly suggest that cyclin E2 turnover can be mediated in a manner that is independent from the mechanisms previously established to control cyclin E1 expression.

Previous studies have often concluded that the roles of cyclin E1 and cyclin E2 are redundant. Nevertheless, Keyomarsi group have recently demonstrated that full length cyclin E1 protein, but not cyclin E2, can be cleaved [26] and that the low molecular weight form of cyclin E1 can play an important role in tumorigenesis. In addition, Musgrove group have lately reported that cyclin E2 influences genomic instability via mechanisms that are distinct from cyclin E1 [27]. In addition, the present work demonstrated that stimulation of hERG1 channel leads to activation of a degradation pathway for cyclin E2 that is distinct from cyclin E1. These reports suggest that, despite their high sequence homology, the function and regulation of type E cyclins may not completely overlap.

Interestingly, it has been reported that cyclin E2 can be an independent and better prognostic marker for some breast cancer subtypes (including luminal A and HER2 positive) when compared with cyclin E1. In addition, cyclin E2 amplification has been liked to breast cancer therapeutic resistance. For example, breast cancers that have become resistant to anti-estrogen therapies (that inhibit cyclin D/CDK4 activity) present an amplification of cyclin E2, suggesting that the presence of cyclin E2 could be a potential mechanism of endocrine resistance. In addition, endocrine resistance has been demonstrated to be overcome by exposure to CDK2 inhibitors, suggesting that cyclin E2 function is a key component in endocrine therapy resistance. To the best of our knowledge, specific cyclin E2 antagonists have not yet been developed. Our study for the first time demonstrated that, stimulation of the hERG1 channel activity rapidly suppresses cyclin E2 in diverse breast cancers cell lines including Trastuzumab-resistant cells.

In conclusion, our study describes an unprecedented mechanism of cyclin E2 degradation in breast cancer cells which contributes to understanding of an important role of cyclin E2 in breast cancer biology. Taken together, these data suggest that pharmacological stimulation of hERG1 potassium channel could be considered as potential therapeutic approach for treating breast cancers that may enhance the effectiveness of current drugs in overcoming therapeutic resistance.

## MATERIALS AND METHODS

### Cell culture

SKBr3 cells were cultured in RPMI-1640 (Thermo Scientific) containing 10% FBS (Serum Source International) and 100 μl/ml penicillin/streptomycin (Thermo Scientific). MCF7 cells were cultured in DMEM (Corning) containing 10% FBS and 100 μl/ml penicillin/streptomycin and 1x NEAA (Thermo Scientific). All cells were grown at 37°C in 5% CO2. The Trastuzumab-resistant SKBR3 cells were a gift from Dr. Clodia Osipo at Loyola University Chicago, Cardinal Bernardin Cancer Center.

### siRNA transfection

SKBr3 cells were transfected with 50 nM of small interfering RNA (siRNA) specific for cyclin E2 (Santa Cruz Biotechnology; sc-37595) using 6 μL Hyperfect transfection reagent (Quiagen) following the manufacturer's instructions. The efficiency of siRNA cell transfections was validated using anti-cyclin E2 Western blotting.

### Western blot

SKBr3 cells were harvested for western blot analysis by trypsinization with 0.25% trypsin-EDTA (Thermo Scientific). Cells were washed with 1x PBS (Thermo Scientific) and resuspended in cold radioimmunoprecipitation assay buffer (RIPA) 50 mM Tris HCl (pH 8.0), 150 mM NaCl, 1% Tergitol (Sigma-Aldrich, St. Louis, MO, USA ), 0.5% Na-deoxycholate, 0.1% SDS, 1 mM phenylmethylsulfonyl fluoride, 1 mM NaF, 1 mM Na3VO4, and 1x Halt Protease Inhibitor Cocktail (Thermo Scientific). Protein concentration was determined by BCA assay (Thermo Scientific). 4x Laemmli buffer containing 10% 2-mercaptoethanol was added to lysates and boiled at 95°C for 5 minutes. Lysates were loaded on 8% SDS-PAGE gels and transferred onto nitrocellulose membranes (Bio-Rad). Membranes were blocked in 5% non-fat milk in mixture of tris-buffered saline containing 0.1% Tween 20 (Sigma-Aldrich) (TBST). Membranes were probed with antibody overnight. Antibodies were resuspended in 5% BSA with 0.01% sodium azide. Antibodies for cyclin A, cyclin B, cyclin E2 were diluted 1:1000 and B-actin was diluted 1:8000 (Cell Signalling Technologies). Antibodies for cyclin E1 and ubitquitin (Santa Cruz) were diluted 1:1000. 370 μg of total cellular protein was used for immunprecipitiation of cyclin E2. Protein was incubated in 4 μL cyclin E2 antibody at 4°C for 4 hours. Rabbit IgG (Santa Cruz) was used as control. Antibody/lysate mixture was incubated with protein A-agarose beads (Roche) at 4°C for 1.5 hours. Lysates were loaded on 8% SDS-PAGE gels and transferred onto nitrocellulose membranes (Bio-Rad).

### Ca2+ imaging and electrophysiology

SK-BR3 cells were seeded onto poly-L-lysine (Sigma)-coated 25 mm glass coverslips. The intracellular free calcium concentration ([Ca2+]i) was measured using digital video microfluorimetry (Tran et al., 2005). The radiometric indicator used was fura-2 (Molecular Probes, Eugene OR). The cells were loaded with fura-2 acetoxymethyl ester (2 μM) for 30 min at room temperature and washed with a balanced salt solution (BSS; containing mM: 145 NaCl, 5 KCl, 2 CaCl2, 1 MgCl2, 10 HEPES and 10 glucose). Sucrose is supplemented to adjust osmolarity of the solution to 200 mOsm. Cells were allowed at least 30 min following washing to allow for dye de-esterification. The glass coverslips were then mounted in a custom-designed sample chamber and perfused with BSS solution at a rate of 1.5 ml/min by a gravity-fed system. Drugs were applied by complete bath exchange using the perfusion system. Free Ca2+ concentration was measured by digital video microfluorimetry using an intensified CCD camera (Hamamatsu). The camera was coupled to a microscope (Nikon Diaphot), and the data acquisition was carried out by a Pentium computer using the MetaFluor software from Universal Imaging.

SK-BR3 cells were seeded onto poly-L-lysine (Sigma)-coated 25 mm glass coverslips (Deutsche Spiegelglas, Carolina Biological supply, Burlinghton, NC, USA). Changes of voltage membrane Vm under current-clamp conditions were monitored 24 hr after seeding by using a HEKA EPC-10 amplifier (HEKA Electronics, Lambrecht/Pfalz, Germany). A fire-polished 1.5 MΩ glass pipettes filled with140 mM KCl, 2 mM MgCl2, and 10 mM HEPES (pH 7.3). Immediately before recording, a 100mg/ml stock of gramicidin was prepared in DMSO and added to the pipette solution (0.5mg/ml) which was then ultra-sonicated and used within 2–3 hr. The standard bath solution contained 140 mM KCl, 0.1 mM CaCl2, 2 mM MgCl2, 10 mM HEPES, and 10 mM glucose (pH 7.3). Only isolated spindle-shaped cells were selected for recording. The value of the membrane potential in the isolated SKBr3 cells presented an average value of −36.8 ± 1.3 mV (*n* = 12).

## SUPPLEMENTARY FIGURES


