# Supplementary material for: Stimulation of hERG1 channel activity promotes a calcium-dependent degradation of cyclin E2, but not cyclin E1, in breast cancer cells
Source: Oncotarget. 2015 Jan 19;6(3):1631–9. doi: 10.18632/oncotarget.2829 (PMC4359320; doi:10.18632/oncotarget.2829)
Supplement: Supplementary file 1 [file oncotarget-06-1631-s001.pdf]

SUPPLEMENTARY FIGURES

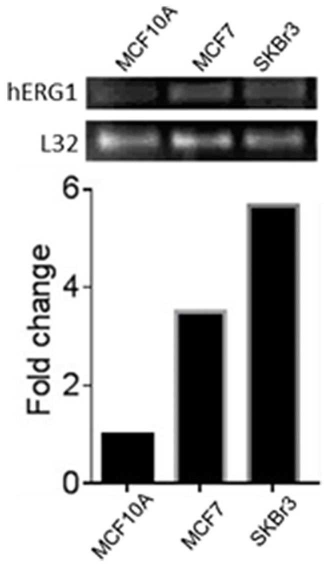

Supplementary Figure 1: hERG1 expression is amplified breast cancer cell lines but not in non-transformed breast cells MCF10A. (upper panel). Semi-quantitative RT-PCR for hERG1 expression in breast cancer cell lines (MCF7 and SKBr3) and in MCF10A non-tumorigenic mammary epithelial cells. L32 expression was utilized as a control (lower panel). The bar graph depicts fold expression derived from densitometry of the observed hERG1 bands normalized to L32 expression. The primer design and cycling conditions were adapted from the report published by Crociani and colleagues [29]

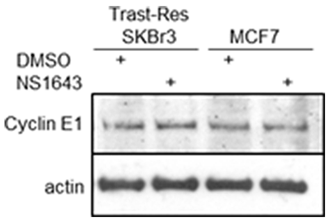

Supplementary Figure 2: NS1643 does not exert any effect on cyclin E1 protein level in Trastuzumab-resistant cells or Luminal A cells. Representative anti-cyclin E1 immunoblot analysis of Trastuzumab-resistant SKBr3 cells and MCF7 cell lysates. The cells were exposed to NS1643 (50 µM) for 4 hr. Cell lysates were resolved by SDS-PAGE.

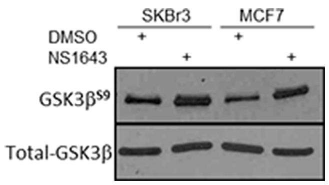

Supplementary Figure 3: NS1643 inhibits GSK3b function. Representative immunoblots of lysates extracted from SKBr3 or MCF7 cells exposed to NS1643 (50 µM) for 4 hr. Immunblots performed with anti-phospho-GSK-3β (Ser9) and anti-GSK-3β antibody are shown.
